# Plasmon-enhanced Raman spectroscopic metrics for *in situ* quantitative and dynamic assays of cell apoptosis and necrosis†
†Electronic supplementary information (ESI) available: Additional experimental details, UV-vis absorption spectrum of AuNSs, dark field scattering spectrum of HSC-3 cells with NT-AuNSs in nucleus, configuration of PERS setup, control Raman spectrum of NT-AuNTs, calculation results according to different Raman bands, and PERS spectroscopy of HSC-3 and MCF-7 cells. See DOI: 10.1039/c6sc02486f



**DOI:** 10.1039/c6sc02486f

**Published:** 2016-10-03

**Authors:** Bin Kang, Shan-Shan Li, Qi-Yuan Guan, Ai-Ping Chen, Pan-Ke Zhang, Li-Bin Zhang, Ji-Wu Wei, Jing-Juan Xu, Hong-Yuan Chen

**Affiliations:** a State Key Laboratory of Analytical Chemistry for Life Science , Collaborative Innovation Center of Chemistry for Life Sciences , School of Chemistry and Chemical Engineering , Nanjing University , 210023 , China . Email: hychen@nju.edu.c ; Email: xujj@nju.edu.cn; b Jiangsu Key Laboratory of Molecular Medicine , Medical School , The State Key Laboratory of Pharmaceutical Biotechnology , Nanjing University , 210093 , China

## Abstract

Apoptosis and necrosis are distinct cell death processes related to many cellular pathways. *In situ*, quantitatively and dynamically monitoring such processes may provide vitally important information for cell studies. However, such a method still remains elusive, even though current immunochemical methodologies have developed extremely valuable tools. Herein, we demonstrate Raman spectroscopic metrics for validating and quantifying apoptotic and necrotic cells based on their distinct molecular vibrational fingerprints. It not only allows us to quantify apoptotic and necrotic cell populations *in situ* in adherent cell samples, but also to be capable of continuously monitoring the dynamical processes of apoptosis and necrosis at the same time in one sample. This method provides comparable results with the “gold standard” of flow cytometry, moreover, with several incomparable advantages. Our work offers a powerful new tool for cell apoptosis and necrosis assays and is expected to become a benchmark technology in biological and medical studies.

## Abstract 2

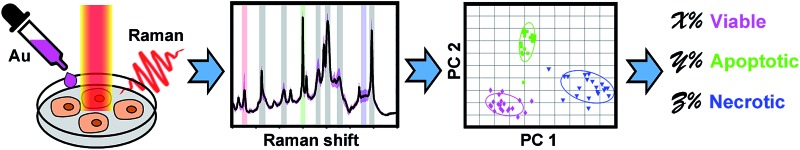
Plasmon-enhanced Raman spectroscopic metrics were developed for *in situ* quantitative and dynamic assays of viable, apoptotic and necrotic cells.

## Introduction

Cell apoptosis and necrosis are very critical biological processes related to many fields of biological and medical research.^1–4^ The apoptotic and necrotic cell populations and related molecular pathways could be identified by several well-established methodologies, including flow cytometry and microscopy based on immunofluorescence labeling,^5,6^ DNA electrophoresis,^7,8^ Western blotting,^9,10^ enzyme colorimetric assay^11^ and so on. However, *in situ*, quantitatively and dynamically monitoring such different cell death processes still remains a great challenge. For instance, flow cytometry requires suspending cells in a stream of fluid, thus it is not available for *in situ* testing of adherent cells and it cannot follow dynamics on the same cell sample. Microscopic imaging based on immunofluorescence is capable of *in situ* validation of apoptotic or necrotic cells; however, it is still hard to provide high-throughput quantitative data using current microscopy techniques in the way that flow cytometry does. Other molecular biological methodologies such as DNA electrophoresis, Western blotting and enzyme colorimetric assays are also not capable of *in situ* testing of living cells since such methods require collecting molecules from cell lysis.^7,8,10,11^

Over the past decades, Raman spectroscopy has been intensively explored for the investigation of molecular constitutions within cells.^12–20^ As a non-invasive optical technique, Raman spectroscopy allows continuous analysis of chemical information inside living cells.^15,17,19,21^ Various techniques, including coherent anti-Stokes Raman spectroscopy (CARS),^15,22,23^ stimulated Raman spectroscopy (SRS),^12,16^ and tip or surface-enhanced Raman spectroscopy (TERS or SERS)^24–29^ have been developed. Particularly, combined with the targeting ability of bio-conjugated plasmonic nanoparticles, the spectral features from specific regions (*e.g.* the nucleus) of single living cells can be detected by plasmonic-enhanced Raman spectroscopy (PERS),^30–34^ which enable the monitoring of molecular events during the cell cycle,^35,36^ cell apoptosis^37,38^ and intracellular drug action,^39,40^ at the single-cell level. However, single-cell based Raman spectroscopy collects the spectrum of one cell at a time. Since Raman scattering from a single cell is mostly weaker than fluorescence, it thus requires a much longer time to integrate the signals. Such distinct limitations were considered to be the main hurdle for single-cell Raman spectroscopy to achieving high-throughput screening. Collecting the mixed spectra of a large number of cells simultaneously would definitely increase the throughput, however; extracting and quantitating the spectral features from multi-component samples became another challenge. Fortunately, it was possible to address such challenges by applying chemometric methods, such as principal component analysis (PCA)^25,41^ and so on.

Herein, combining PERS and chemometrics together, we present a Raman metric strategy for an *in situ* quantitative and dynamic assay of apoptotic and necrotic cells based on measuring their distinct molecular signatures. Through analyzing the PERS spectra from the biomolecules at the cell nucleus, the correlated Raman bands of viable, apoptotic and necrotic cells were identified and different cell populations were clearly discriminated. We then developed a mathematical model to calculate the percentage of viable, apoptotic and necrotic cell populations from the averaged PERS spectra of adherent cell samples. This strategy not only allows *in situ* quantitative testing of apoptotic and necrotic cells, but is also capable of monitoring the dynamic apoptotic and necrotic processes in the same cell sample over a different period of time. Compared to the current “gold standard” flow cytometry, our PERS strategy could provide pretty consistent results, with an average difference of 2.86%, moreover, it has several incomparable advantages. Firstly, it works for an adherent cell sample. Secondly, it allows *in situ* measurement of living cells. Thirdly, it enables the study of the dynamics of cell death in real time. This technique provides a new tool for *in situ*, quantitative and dynamic analysis of cell apoptosis and necrosis directly in adherent cell samples, which may promote cell analysis applications in many fields.

## Results and discussion

### Readout Raman spectra from cells

**Fig. 1:**
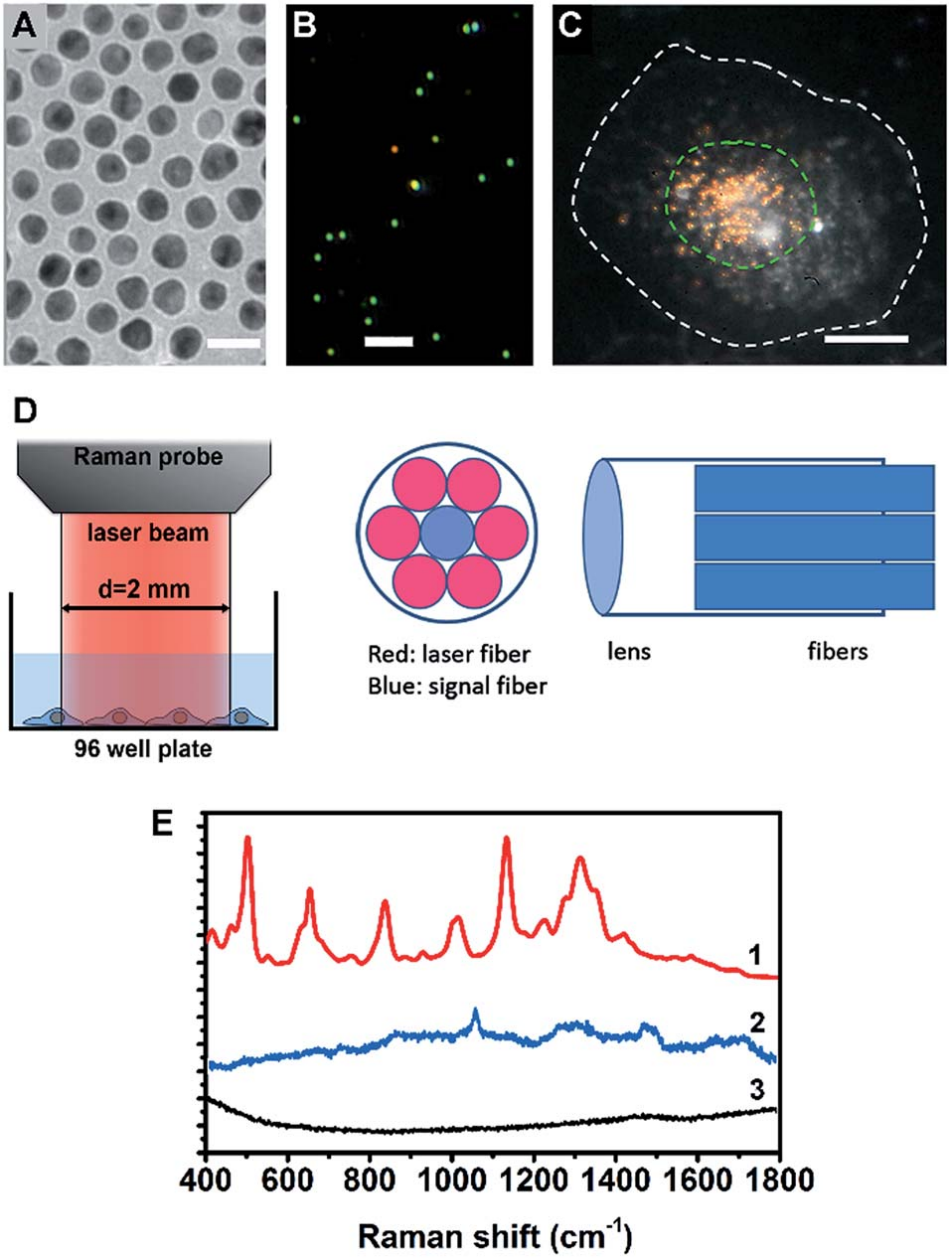
(A) TEM image of the AuNSs, the scale bar is 20 nm. (B) Dark field scattering image of the AuNSs, the scale bar is 2 μm. (C) Dark field image of a HSC-3 cell pre-incubated with nuclear-targeted gold nanospheres (NT-AuNSs), the NT-AuNSs are mainly localized at the nuclear region. The white dashed line indicates the cell boundary and the green dashed line indicates the cell nucleus, the scale bar is 10 μm. (D) The optical configuration of the Raman probe, the laser beam from the Raman probe is about 2 mm in size, which roughly covers one well of a 96-well plate. (E) Typical Raman spectra of cells with (1) and without (3) NT-AuNSs, and the spectrum of NT-AuNSs (2).

A cell nucleus could be considered as the most important subcellular compartment since it contains most of the DNA and various proteins within cells. The spontaneous Raman signals from these biological molecules are usually very weak because of their low scattering cross section. To amplify the Raman signals of DNA/protein complexes at the cell nucleus, we utilized nuclear-targeting gold nanospheres (NT-AuNSs). The NT-AuNSs were synthesized *via* a citrate-reduction approach, and were further conjugated with methyl polyethylene glycol (mPEG), arginine–glycine–aspartic acid (RGD) and nuclear localization signal (NLS). These NT-AuNSs were about 20 ± 4 nm in diameter (Fig. 1A). They have a plasmon band maximum at 521 nm (Fig. S1, ESI†) in solution, and show bright green Rayleigh/Mie scattering under a dark-field (DF) microscope (Fig. 1B). Once the NT-AuNSs were internalized into cells and aggregated around the nuclear region (Fig. 1C), their plasmon band shifted to 600–800 nm (Fig. S2, ESI†), due to plasmon coupling and Fano-like resonance.^42–44^ The red scattering from aggregates (*i.e.* coupling modes) mixed with the green scattering from individual particles (*i.e.* single-dipole mode), exhibited a bright “golden” color under the DF microscope (Fig. 1C). Such a red-shifted and broadened plasmon band allows us to use a red laser (785 nm) as the incident light for Raman measurements. The Raman spectra were taken on a lab-built Raman plate reader (Fig. S3, ESI†). The laser beam was expand to ∼2 mm in diameter, which roughly covers one well of the 96-well plates (Fig. 1D). This configuration differs with the setup for single cell Raman studies, in which the laser beam was usually tightly focused to ∼1 μm in size.^35,38–40^ Herein, instead of taking the spectra of individual cells one by one, the averaged Raman spectra of a large number of adherent cells (roughly 10^4^ to 10^5^ cells depending on the cell density in wells) were collected, which significantly extended the possibility of reaching high-throughput screening. The presence of NT-AuNSs enhanced the Raman signals of cells ∼10^4^ fold, but none of the Raman bands of the NT-AuNSs appear as strong bands in the cell spectrum (Fig. 1E). The spectral stability of NT-AuNSs was also carefully checked under cellular experimental conditions (Fig. S4A, ESI†), to make sure that the observed changes in the cell spectra were not originating from the nanoparticles.

### Spectroscopic reference samples

**Fig. 2:**
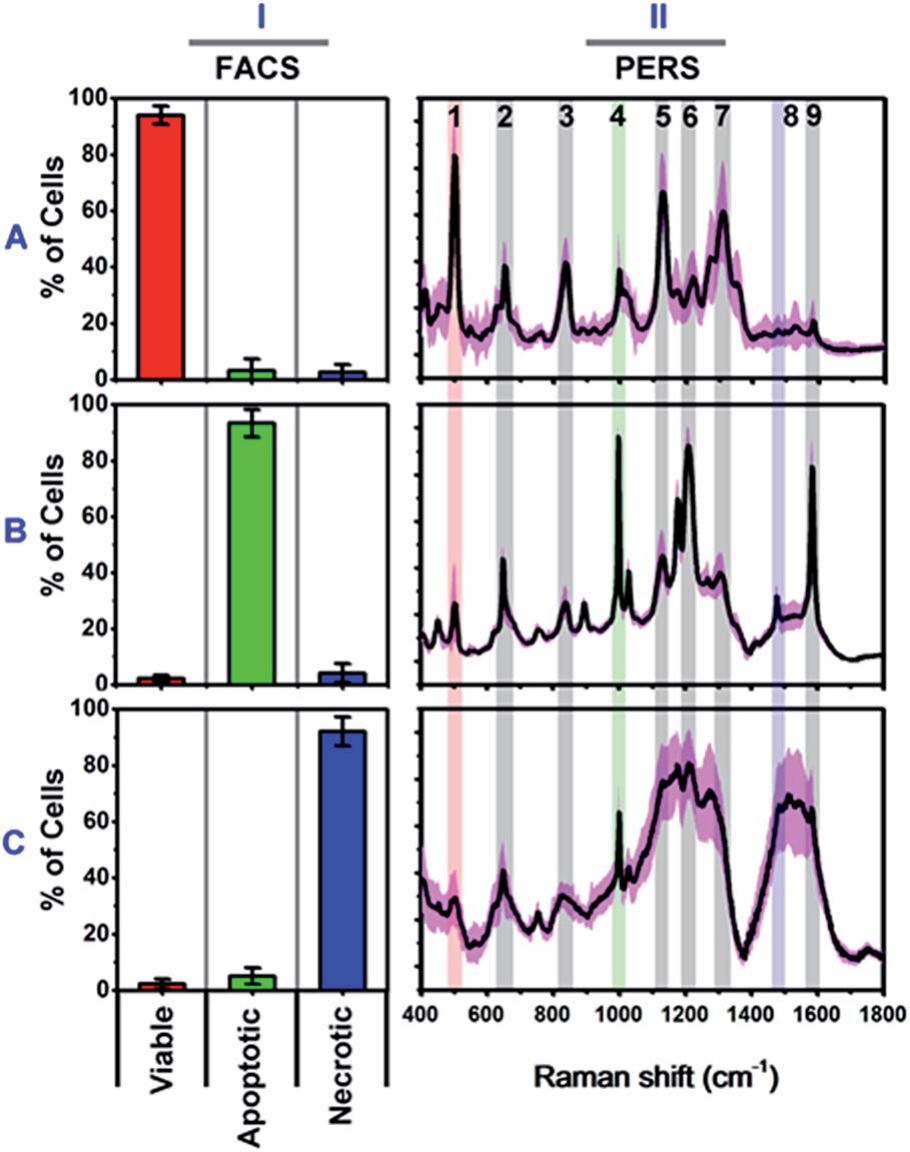
Characterized flow cytometry (FACS) (I) and plasmonic-enhanced Raman spectra (PERS) (II) of reference viable (A), apoptotic (B) and necrotic cells (C). The black lines in the PERS spectra show the averaged spectra from 30 trials of measurements, and the pink colour indicates their standard deviation (*n* = 30). The red (1), green (4) and blue (8) colour in the PERS spectra indicates the most representative bands in each population.

We firstly carried out a set of control experiments to create spectroscopic references of viable, apoptotic and necrotic cells, using HSC-3 cells as a model (see Methods section for details). All cell samples were pretreated with NT-AuNSs for 24 h at a minimum concentration of 0.05 nM, which has been proven to scarcely affect cell viability in the previous literature^38^ as well as in our current study (Fig. S4B, ESI†). The reference viable, apoptotic and necrotic samples were evaluated using flow cytometry, and the percentage of cell populations are 94.02% viable cells, 93.42% apoptotic cells and 92.16% necrotic cells, respectively (Fig. 2I). The PERS spectra of viable, apoptotic and necrotic cells show surprising different features (Fig. 2II). The characteristic bands and their tentative assignments are summarized in Table 1. Viable cells have several characteristic bands at 490–510, 620–660, 820–860, 1000–1020, 1120–1140 and 1280–1320 cm^–1^ (Fig. 2A-II), which could be assigned to the disulfide bonds (S–S),^45–48^ C–S bonds,^48–51^ DNA backbone (O–P–O)/tyrosine,^52–54^ phenylalanine ring breathing,^18,48,55^ peptide bonds (C–N)^48,56–59^ and protein amide III band,^48,58–60^ respectively.

**Table 1:** Assignments of the characterized bands in PERS spectra

	Bands (cm^–1^)	Component	Tentative assignments of PERS bands
1	490–510	Protein	S–S stretching
2	620–660	Protein	C–S stretching
3	820–860	DNA/protein	O–P–O backbone, tyrosine
4	1000–1020	Protein	Ring breathing of phenylalanine
5	1120–1140	Protein/carbohydrate	C–N stretching, C–O/C–C stretching
6	1200–1240	Protein	Amide III (β-pleated sheet)
7	1280–1320	Protein	Amide III (α-helix)
8	1460–1500	Lipid/protein	CH_2_/CH_3_ deformation, bending
9	1560–1600	DNA/protein	Guanine/adenine, phenylalanine

Apoptotic cells have several notable spectroscopic changes compared to viable cells (Fig. 2B-II). Firstly, the bands at 490–510 cm^–1^ decrease, while the bands at 1000–1020 and 1200–1240 cm^–1^ increase. The disulfide (S–S) bonds and C–S bonds play an important role in the folding and stability of protein native structure.^61,62^ In apoptotic cells, the decrease of the S–S band indicates disulfide bond breakage within intracellular proteins when they undergo denaturation.^1,2,4,8,63^ The parallel increase in the 1000–1020 and 1200–1240 cm^–1^ bands also indicates protein denaturation as these bands originate from the ring breathing of phenylalanine, β-pleated sheet conformation or C–C_6_–H_5_ stretching of phenylalanine and tryptophan, which are no longer positioned within the interior of a folded protein.^48,58,61,62^ Secondly, a pronounced decrease in the intensities of the 1120–1140 and 1280–1320 cm^–1^ bands was also seen after apoptotic treatment. Since these two bands are attributed to protein peptide bond (C–N) stretching and α-helix protein structure, thus here, the decrease of these vibration bands indicates that the peptide bond hydrolyses during nuclear protein degradation.^64–66^ Thirdly, the band intensity at 820–860 cm^–1^ decreases while that of the 1560–1600 cm^–1^ band increases. These two bands could be attributed to the DNA O–P–O backbone and ring mode of guanine and adenine, respectively.^52–54,59^ These vibrational changes indicate DNA backbone breakage and the exposure of purine bases, which correlates with nuclear DNA fragmentation as a result of cell apoptosis.^7,8,63,67^ All of the above Raman vibrational changes indicate the occurrence of protein denaturation, protein degradation and DNA fragmentation, which are typical molecular events of cell apoptosis.^1,2,4,8,52^

The Raman features of necrotic cells are different from those of apoptotic cells (Fig. 2C-II). Compared with viable cells, the S–S band at 490–510 cm^–1^ also decreases in necrotic cells, indicating the breakage of disulfide bonds in the protein tertiary structure. However, the peptide bond vibration at 1120–1140 cm^–1^ did not decrease as much as in apoptotic cells, and the phenylalanine band at 1000 cm^–1^ also did not increase so much. The above results indicate that the breakage of disulfide bonds indeed occurs in apoptotic cells, but protein degradation is not as notable as in apoptotic cells. The probable reason is that, for necrotic cells, cell death occurs rapidly, and the structure of enzymes was also damaged in short time, thus the protein cannot undergo proteolysis since there is a lack of active enzymes. Thereby, the necrotic cells do not show as much change in the 1000 cm^–1^ phenylalanine band and 1120–1140 cm^–1^ peptide bond vibration band as apoptotic cells do. The decrease in the intensity of the DNA backbone band at 820–860 cm^–1^ and the increase of the 1560–1600 cm^–1^ band intensity exhibits a similar trend as with the apoptotic cells, which indicates the occurrence of DNA fragmentation after necrotic treatment. In addition to the above changes, the most notable change is the increase of lipid or protein CH_2_/CH_3_ deformation and bending band around the 1460–1500 cm^–1^ region.^19,21,53,68^ The strong lipid vibration in necrotic cells could be attributed to the damage of cell membranes and intracellular vessel structures, thus the NT-AuNSs are no longer located around the cell nucleus and thereby may have got in contact with the cellular membrane and enhanced the lipid signals.

### Discrimination of cell populations

**Fig. 3:**
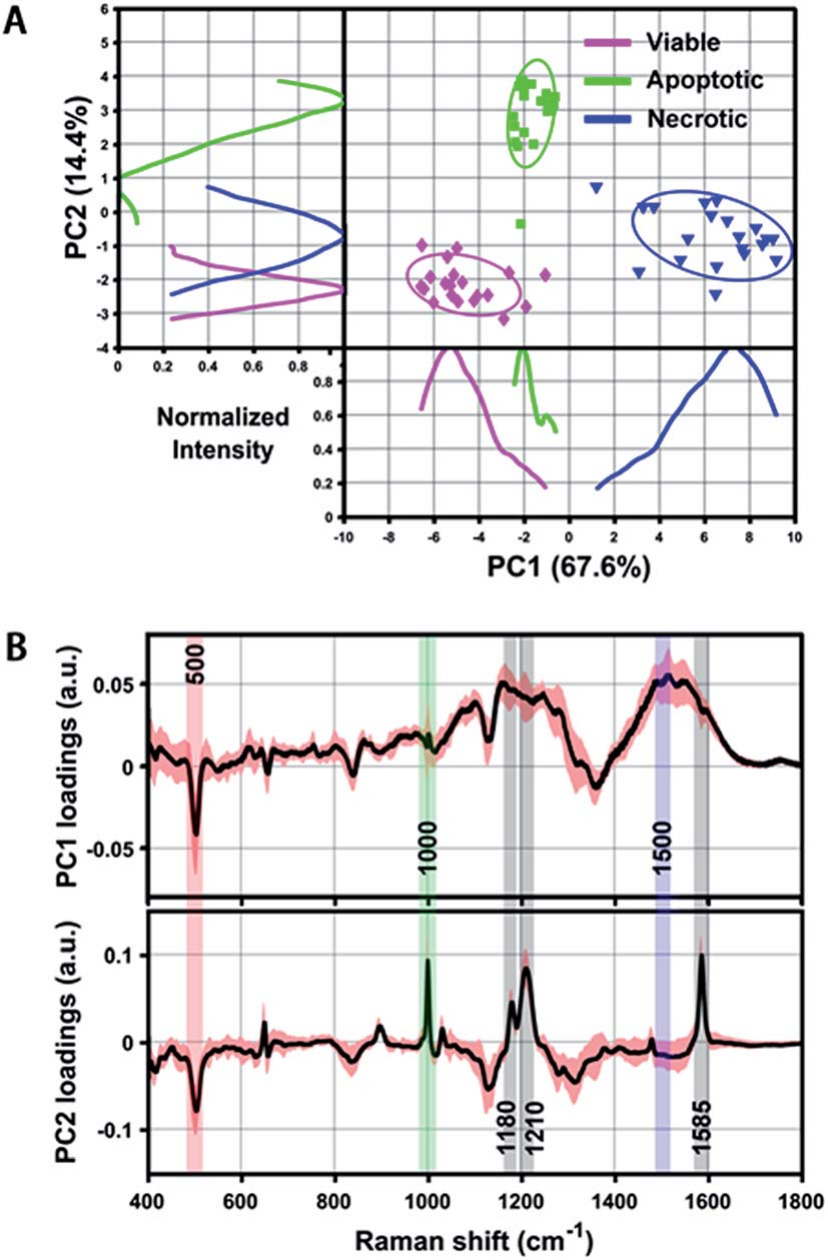
(A) Scatter plot and 1D intensity distribution of PC1 *vs.* PC2 scores originating from the PERS spectra of reference viable, apoptotic and necrotic cell samples. Different cell populations are clearly segregated by either the scatter plot or 1D intensity line of the PCs. Intensity curves were generated by using Kernel density estimation to smooth PC score histograms. (B) PC1 and PC2 loading of all extracted spectra of the above three reference samples, which shows distinct peaks corresponding to the discrimination weight between these cell groups at a certain Raman shift. The averaged PCs from 20 spectra are shown as a black line and their standard deviation is highlighted in red.

Even though we have seen that the viable, apoptotic and necrotic cells exhibit different PERS spectroscopic features, it is still not easy to pick up the most significant differences between each cell population. To address this problem, we utilized principal component analysis (PCA), a chemometric method suitable for extracting principal information from multidimensional data sets. Fig. 3 shows the score plots and loading of the derived principal components (PCs). From the score plots of PC1 and PC2, the classification of viable, apoptotic and necrotic cell populations can be clearly distinguished (Fig. 3A). The loading of PC1 and PC2 reflects the contribution of each variable to the variation of the data set between groups (Fig. 3B). PC1 loading shows peaks at 500 and 1500 cm^–1^ and PC2 loading shows peaks at 500, 1000, 1180, 1210 and 1585 cm^–1^. We also did a difference analysis on the representative Raman features to reveal their correlation to each cell population (see Methods section for details). The representative viable, apoptotic and necrotic features after difference analysis treatment are shown in Fig. S5 in the ESI,† and the analysis on their intensity and correlation is summarized in Table S1 in the ESI.†

### Mathematical model and calculation

Considering overall the band intensity and representativity (Fig. 2II, 3B and S5, Table S1, ESI†), we chose three PERS bands at 500, 1000 and 1500 cm^–1^ to represent the viable, apoptotic and necrotic cell populations, respectively. We then developed a mathematical model to calculate the percentage of viable, apoptotic and necrotic cell populations from the averaged PERS spectra of any given samples. Three ideal samples are assumed, which contain 100% viable cells, 100% apoptotic cells and 100% necrotic cells, named *S*_via_, *S*_apo_ and *S*_nec_. The intensity of characterized Raman bands for these ideal samples are represented by *I*_via(*k*)_, *I*_apo(*k*)_, and *I*_nec(*k*)_ at a specific wavenumber, *k*, since the intensity of averaged Raman signals is proportional to the number of cells. Thus, for a given cell sample termed *S*_mix_, which contains an unknown *x*% of viable cells, *y*% of apoptotic cells and *z*% of necrotic cells, the following equation works:*I*_via(*k*)_ × *x*% + *I*_apo(*k*)_ × *y*% + *I*_nec(*k*)_ × *z*% = *I*_mix(*k*)_where *I*_mix(*k*)_ is the Raman band intensity of a given sample, *S*_mix_, at a specific wavenumber, *k*, and the unknown numbers *x*, *y* and *z* are under the constraint condition of *x* + *y* + *z* = 100.

As mentioned before, the three reference samples used in the current work were experimentally created, and the percentages of viable, apoptotic and necrotic cells were 94.02%, 93.42% and 92.16%, respectively. Since such reference samples are actually not 100% ideal samples, so the Raman band intensity of the above reference samples are not exactly the same as the ideal theoretical samples. To obtain more accurate calculation results, we did a correction on the Raman band intensity of the experimental reference samples by using a linear equation:*I*_ideal_ × (*x*, *y*, *z*)% + *I*_background_ = *I*_reference_where *I*_ideal_ is the theoretical band intensity of the ideal samples, *I*_reference_ is the experimentally observed band intensity of the reference samples in the current work, (*x*, *y*, *z*)% is the percentage of viable, apoptotic and necrotic cells in experimental reference samples, and *I*_background_ is the intensity of nonspecific background at specific wavenumbers.

### Quantifying cell populations

Using the above equation and the corrected *I*_ideal_ of the bands at 500, 1000 and 1500 cm^–1^, we calculated the percentage of viable, apoptotic and necrotic cell populations. As a proof of concept, we tested this method on two adherent cultured cell lines (HSC-3 and MCF-7 cells), under the treatment of four types of anticancer drugs; cisplatin (CisPt), fluorouracil (5-FU), camptothecin (CAMP) and doxorubicin (DOX).

**Fig. 4:**
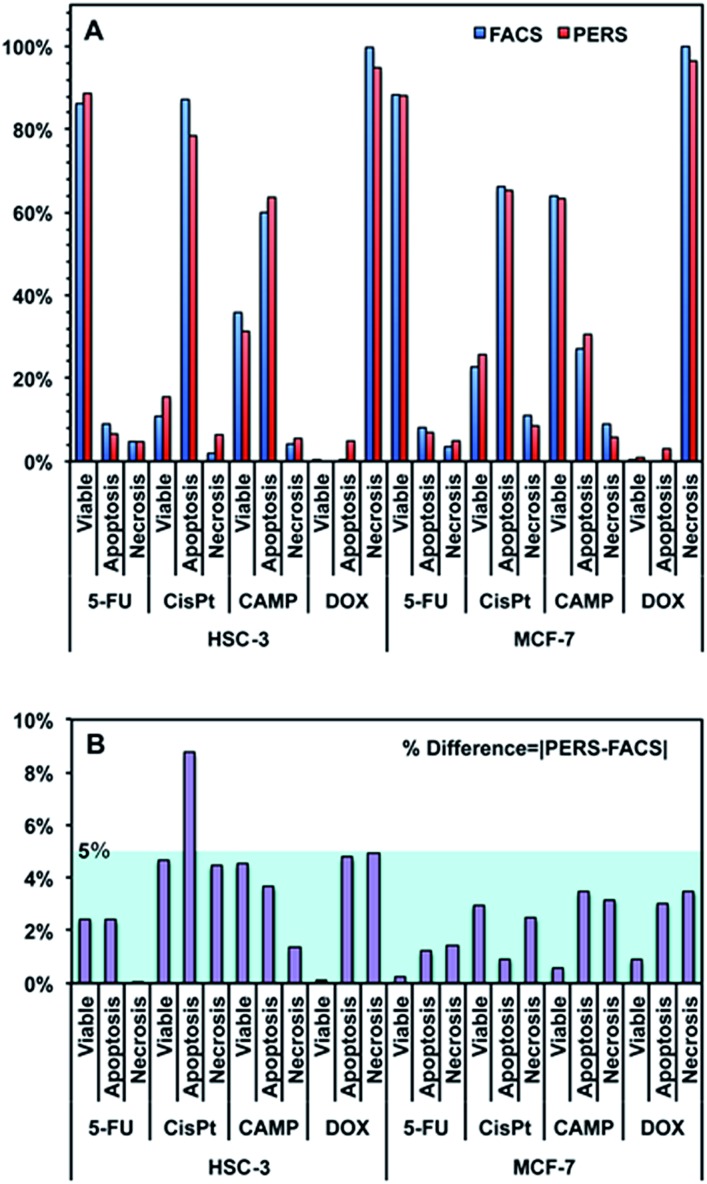
(A) Comparison between the results of viable, apoptotic and necrotic cells measured using flow cytometry (FACS) and PERS. HSC-3 and MCF-7 cells were treated with 100 μM fluorouracil (5-FU), cisplatin (CisPt), camptothecin (CAMP) and doxorubicin (DOX) for 24 h. (B) % difference between the results of PERS and FACS. The % difference is defined as the absolute value of [PERS-FACS].

**Fig. 5:**
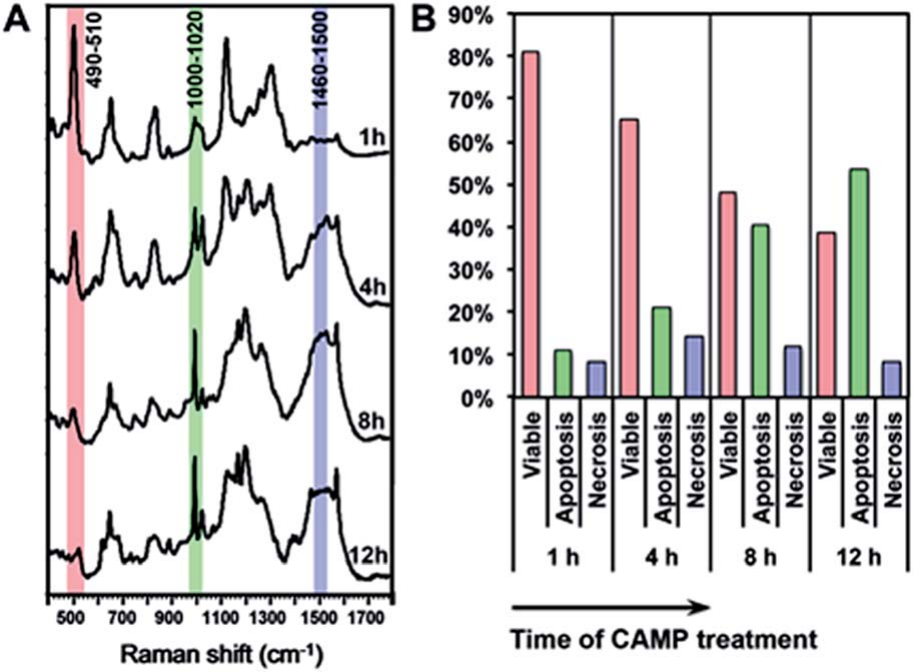
*In situ* dynamic measurement of the viable, apoptotic and necrotic populations of HSC-3 cells. (A) The PERS spectra of HSC-3 cells treated by camptothecin (CAMP) for 1, 4, 8 and 12 h. (B) Percentages of viable, apoptotic and necrotic populations along with the time of CAMP treatment.

Fig. 4 shows the comparison between the results calculated from PERS spectra and the control results from flow cytometry. The results for cell populations obtained by PERS and flow cytometry are very consistent with one another (Fig. 4A). Comparing 24 groups of data pairs, 23 pairs of data obtained by the two above methods have a difference <5%, except for one pair of data with a difference <10% (Fig. 4B). All the 24 pairs of data have an average difference of 2.86% and a coincidence index of >95%. These results suggest that this PERS strategy could provide comparable results with the “gold standard” of flow cytometry. Moreover, this PERS technique allows real-time dynamic measurement on the same cell sample. Fig. 5 shows an example of HSC-3 cells treated by CAMP for different periods of time. PERS spectra from the same cell sample were collected *in situ* at 1, 4, 8 and 12 hours of drug treatment (Fig. 5A). According to the PERS spectra and our proposed mathematical model, the percentages of viable, apoptotic and necrotic cell populations at each time point could be obtained (Fig. 5B). From the time-dependent data, one can clearly see the changes of each cell population along with the time of drug treatments, which is usually not able to be obtained by flow cytometry. It is important to note that the selection of Raman bands for calculation was based on two criteria: (1) the band intensity should be strong and (2) the band should appear in one cell population, but not in the other two. According to the above criteria and the analysis in Fig. S5 and Table S1,† the 500, 1000 and 1500 cm^–1^ bands were selected for calculating the cell populations. Calculations according to other bands were also tried (see Fig. S6 and S7 in the ESI†), but in our current system, the calculation results according to 500, 1000 and 1500 cm^–1^ bands were the best, which also confirms the feasibility of band selection criteria.

While PERS spectroscopy provided comparable results about cell apoptosis and necrosis with flow cytometry, they are based on different mechanisms. Different from flow cytometry using immunofluorescence labeling, the PERS spectroscopy distinguish different cell populations according to their distinct molecular signatures. As a proof of principle demonstration, the current study did not focus on distinguishing the different stages of apoptotic cells, *i.e.* early or late apoptosis. Actually, the plentiful spectral features in Raman spectroscopy make it possible to not only distinguish the early and late stages of cell apoptosis, but also to reveal the molecular kinetics during apoptotic processes.^38,39^ A full understanding of the detailed molecular events, dynamics and mechanisms about the cell death process was expected to be revealed *via* monitoring more molecular vibrational features and utilizing more integrated spectral analysis.

In present study, we utilized two types of adherent cultured cell lines (HSC-3 and MCF-7 cells) as models. These two types of cells exhibit very similar PERS spectroscopic features when they are treated using the same viable, apoptotic and necrotic procedures for creating reference samples (Fig. S8–S10, ESI†). Most of the characteristic bands of these two types of cells are very consistent in terms of both band position and intensity. The reason could possibly be that in our current work, nuclear-targeting Au-NSs were utilized to enhance the Raman signals of biomolecules at the cell nucleus that mainly contains DNA/protein complexes, and thus the obtained Raman spectra are mainly contributed by the vibrational modes of DNA and protein molecules. As we know, a cell nucleus consists of more than one type of DNA and protein molecules, and their sequence and detailed high-order structure may depend on the types of cell. However, all the protein and DNA molecules contain the same general chemical structures, such as S–S and C–S bonds, peptide C–N bonds, DNA O–P–O backbone bases, amino acids and so on. Thus these general chemical structures contribute strong Raman signals and lead to the similarity of the PERS spectra of HSC-3 and MCF-7 cells. This suggests that the PERS strategy has potential to be a universal methodology for measuring the apoptosis and necrosis of various types of cells. It is notable that even the PERS spectra of HSC-3 and MCF-7 cells are similar to their reference samples, the Raman bands change with different profile and dynamics when they undergo apoptotic or necrotic cell death (Fig. 4A). As a final remark, the PERS spectral features of cells may depend on the design of plasmonic nanoparticles. We utilized nuclear-targeting gold nanospheres in the current work, however, the plasmonic nanoprobes did not have to be spherical in shape, and they did not have to target to the cell nucleus. In principle, nanoparticles with different shapes or different targeting conjugations should also work. The use of different nanoparticles might result in different Raman spectra features and different selection of representative bands for calculating cell populations. The strategy of spectroscopic metrics in the current work, such as spectra analysis, calculation and mathematical model, could be generalized for all types of nanoparticles.

## Conclusions

In conclusion, we report a Raman spectroscopic metric approach for *in situ*, quantitative and dynamic testing of apoptotic and necrotic cell death. Compared to the current “gold-standard” flow cytometry, this spectroscopic technique has several potential advantages: firstly, unlike flow cytometry that requires cells in suspension, it also works for adherent cell samples, secondly, it allows *in situ* measurement of living cells, and thirdly not only as an end-point test; it enables the study of the dynamics of cell death in real time. Although this technique in our current work is still in its infancy, it makes it possible to measure cell apoptosis and necrosis *in situ*, quantitatively and dynamically, which may become a benchmark technology in molecular biology. The spectroscopic metric strategy in our current work may shed light on developing spectroscopy-based methods for quantitative cell analysis.

## Experimental section

### Nanoparticles preparation and conjugation

Gold nanospheres (AuNSs) were synthesized using a citrate-reduction based approach,^69^ as reported in the previous literature.^35–40,42^ Briefly, 25 mL of 20 mM trisodium citrate was added to 475 mL of a 1.6 mM HAuCl_4_ solution at 100 °C under stirring, until the color changed to red. Then, the AuNSs were washed with deionized (DI) water *via* centrifugation at 6000 rpm for 15 min. For PEGylation, a 1.0 mM solution of thiol-modified methoxypolyethylene glycol (mPEG-SH, MW 5000) was added to a AuNSs solution with a final molar ratio of 1000 : 1. The PEG–AuNSs solution was incubated for 24 hours, and then centrifuged at 6000 rpm for 15 min and washed with DI water. After PEG modification, the AuNSs were conjugated with RGD and NLS peptides by the addition of a 5.0 mM RGD (CGPDGRDGRDGRDGR) peptide solution and a 5.0 mM NLS (GGVKRKKKPGGC) peptide solution to PEG–AuNSs with a molar excess of 10^4^ and 10^5^, respectively. The RGD/NLS–AuNSs were purified by centrifugation (10 000 rpm, 5 min) to remove unbound ligands. The final obtained nuclear-targeting AuNSs were termed NT-AuNSs.

### Cell culture

Human oral squamous cell carcinoma (HSC-3) and human breast adenocarcinoma (MCF-7) cells were cultured in Dulbecco's Modified Eagle's Medium (DMEM, Mediatech), supplemented with 10% fetal bovine serum (FBS, Mediatech) and 1% antimycotic solution (Mediatech), in a 37 °C and 5% CO_2_ humidified incubator. For PERS experiments, the cells were cultured in a 96-well culture plate and pretreated with 0.05 nM NT-AuNSs for 24 hours before collecting spectra.

### Spectroscopic reference samples

The reference viable cells are control cells without any treatment except for 0.05 nM NT-AuNSs pretreatment; the reference apoptotic cells were induced by 100 μM H_2_O_2_ treatment for 24 h, and the reference necrotic cells were treated in boiling water (100 °C) for 10 min. The viable, apoptotic and necrotic cells were evaluated by flow cytometry using a standard Annexin V-FITC and PI double staining procedure.

### PERS readout

The PERS experiments were carried out on a lab-built Raman plate reader setup (Fig. S3, ESI†). This setup combined a fiber Raman spectroscopy system with a motor driven XY stage. Briefly, a 785 nm laser was used as incident light. The incident laser was guided to a home-modified Raman probe through a multimode Y-shaped fiber. The laser beam output from the Raman probe was about 2 mm in diameter, which roughly covered one well of the 96-well culture plate. The Raman signals from samples were collected with the same Raman probe and delivered to a fiber spectrometer through another arm of the Y-fiber. Typically, with the enhancement of plasmonic nanoprobes, the PERS spectra were acquired with a CCD integration time of 500 ms. The motor driven stage allow us to read out the Raman spectra of each well, under the control of programmed software. For *in situ* dynamic PERS measurement, the stage and culture plate were covered with a homemade case with a temperature control unit and an access to inject 5% CO_2_ to keep the cells alive. For each group, spectra from 10 wells of cells were taken to get an average result, and at least 3 independent trials were taken for all experiments.

### Flow cytometry

Flow cytometry based on Annexin V-FITC and propidium iodide (PI) staining was used to confirm the results of PERS spectroscopy. For this measurement, cells were grown in 12-well culture plates and pretreated with 0.05 nM NLS/RGD–AuNSs for 24 h before any drug treatment. After drug treatments, cells were collected after trypsinization and washed with cold DPBS. Then, cells were redispersed in binding buffer, and 5 μL of Annexin V-FITC and 2 μL of propidium iodide (PI) were added. Cell solutions were then incubated for 15 min at room temperature and run on a BD flow cytometer by using standard FITC and PI channels. Viable, apoptotic and necrotic cell populations were analyzed using FlowJo software.

### Principal component analysis (PCA)

PCA is a suitable tool for extracting principal information from multidimensional data sets such as Raman spectra. In the current study, the PC scores and loadings were calculated *via* Matlab R2015b. Before running PCA, 20 spectra from each group (viable, apoptotic, necrotic) were randomly selected and normalized from 0 to 1. Scatter plots of PC1 and PC2 scores were utilized to classify different cell populations. A Kernel density estimation method is used to smooth the diagram of scores to generate a 1D intensity distribution. PC1 and PC2 loading exhibited the discrimination weight between different cell groups at certain Raman shifts. To address this, three Raman spectra were randomly selected from each of the three cell groups, and a PC loading curve was calculated by running PCA on these three spectra. This calculation process was carried out 20 times to get the average value (black line) and standard deviation (highlighted as red color) of all 20 PC loadings.

### Calculating the percentages of cell populations

The percentage of cell apoptosis and necrosis were calculated according to the linear equation sets proposed in the current work. The equations were solved by using Mathematic software, the algorithm was trained by inputting the reference data of cell apoptosis and necrosis obtained by flow cytometry, and a self-consistent calculation strategy was used to get a convergent numerical solution. Calculations were tried according to the normalized intensity of different bands (Fig. S6 and S7, ESI†), the bands of 500, 1000 and 1500 cm^–1^ gave the best calculation results in our current system.
