# Supplementary material for: Plasmon-enhanced Raman spectroscopic metrics for in situ quantitative and dynamic assays of cell apoptosis and necrosis
Source: Chem Sci. 2016 Oct 3;8(2):1243–50. doi: 10.1039/c6sc02486f (PMC5369531; doi:10.1039/c6sc02486f)
Supplement: Supplementary file 1 [file SC-008-C6SC02486F-s001.pdf]

Supporting Information

Plasmon-enhanced Raman Spectroscopic Metrics for *In situ*, Quantitative and Dynamic Assays of Cell Apoptosis and Necrosis

Bin Kang,[a] Shan-Shan Li,[a] Qi-Yuan Guan,[a] Ai-Ping Chen,[b] Pan-Ke Zhang,[a] Li-Bin Zhang,[a] Ji-Wu Wei,[b] Jing-Juan Xu*[a] and Hong-Yuan Chen*[a]

[a] State Key Laboratory of Analytical Chemistry for Life Science and Collaborative Innovation Center of Chemistry for Life Sciences, School of Chemistry and Chemical Engineering, Nanjing University, 210023, China.

E-mail: hychen@nju.edu.c; xujj@nju.edu.cn

[b] Jiangsu Key Laboratory of Molecular Medicine, Medical School and the State Key Laboratory of Pharmaceutical Biotechnology, Nanjing University, 210093, China

This file includes:

Additional experimental details

Figure S1. The UV-Vis absorbance spectrum of NT-AuNSs in solution.

Figure S2. The dark field scattering spectrum of a HSC-3 cell with NT-AuNSs targeted to its nucleus.

Figure S3. The setup configurations of the Raman plate reader used in current work.

Figure S4. (A)Raman spectra of NT-AuNSs, NT-AuNSs incubated in cell culture medium for 24 h, NT-AuNSs treated by 1 mM H_2O_2 for 24 h and NT-AuNSs heated at 100 °C for 15 min. (B) XTT results cell viability in the presence of 0.05 nM NT-AuNSs at different periods of incubation.

Figure S5. Representative PERS features of the viable (A), apoptotic (B) and necrotic cells (C).

Figure S6. Comparison between results of FACS and PERS calculated according to the intensity of bands at 500, 1000 and 1585 cm^{-1}.

Figure S7. Comparison between results of FACS and PERS calculated according to the intensity of bands at 1180, 1210 and 1585 cm^{-1}.

Figure S8. Plasmonic-enhanced Raman spectra of viable HSC-3 cells (black) and MCF-7 cells (red).

Figure S9. Plasmonic-enhanced Raman spectra of apoptotic HSC-3 cells (blue) and MCF-7 cells (red).

Figure S10. Plasmonic-enhanced Raman spectra of necrotic HSC-3 cells (blue) and MCF-7 cells (red).

Table S1. Analysis on the represented PERS features of viable, apoptotic and necrotic cells.

Additional experimental details:

Raman spectral stability of NT-AuNSs. To ensure that the NT-AuNSs were stable in cell culture environment or under apoptotic and necrotic treatment, a series of control experiments were carried out. We firstly compared the Raman spectrum of NT-AuNSs itself and the spectrum of cells with NT-AuNSs targeted to cell nucleus. None of the Raman features of NT-AuNSs appeared as strong bands in the spectra of cells. We also tested the stability of NT-AuNSs under the same condition of cellular treatment, including incubated in cell culture medium at 37 °C for 24 h, treated by 1 mM (10 folds higher than treating cells) H_2O_2 for 24 h and heated at 100 °C for 15 min (5 min longer than treating cells). None of obvious spectral changes were seen after above treatments, which suggested the stability of the NT-AuNSs used in our current work (Figure S4).

Spectral difference-analysis. In order to reveal the correlation of PERS spectra to cell populations, we did a difference-analysis based on the representative Raman features. The representative feature for each cell populations were defined as:

Viable features=[Via-Apo]+[Via-Nec]

Apoptotic features=[Apo-Via]+[Apo-Nec]

Necrotic features=[Nec-Via]+[Nec-Apo]

in which Via, Apo and Nec are the normalized PERS spectra of viable, apoptotic and necrotic cells. Figure S5 shows the representative viable, apoptotic and necrotic feature after difference-analysis treatment, and the analysis on the intensity and correlation of Raman features was summarized in Table S1.

Applicability to different cell types. In present study, we utilized two types of adherent cultured cell lines (HSC-3 and MCF-7 cells) as models. These two types of cells exhibited very similar PERS spectroscopic features when they treated by the same viable, apoptotic and necrotic procedures for creating the reference cell samples (Figure S8-S10). This result suggested that the PERS strategy is potentially to be a universal methodology for measuring the apoptosis and necrosis of various types of cells.

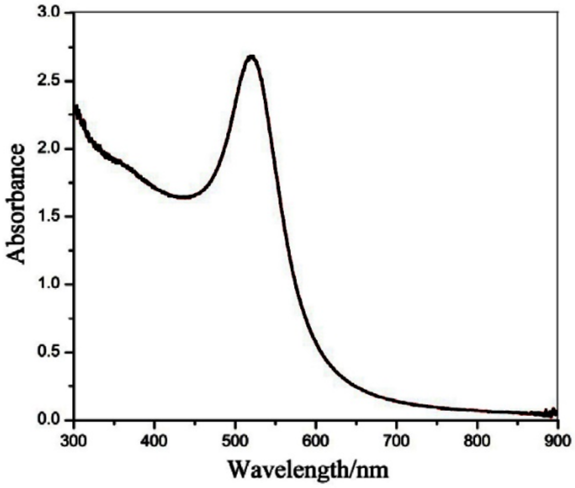

Figure S1. The UV-Vis absorbance spectrum of NT-AuNSs in solution. The NT-AuNSs solution have an absorption band maximum at 521 nm.

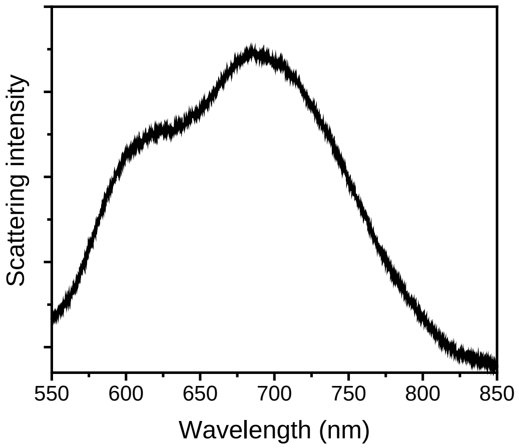

Figure S2. The dark field scattering spectrum of a HSC-3 cell with NT-AuNSs targeted to its nucleus. This broaden and red-shifted band is due to the plasmonic coupling between particle aggregates.

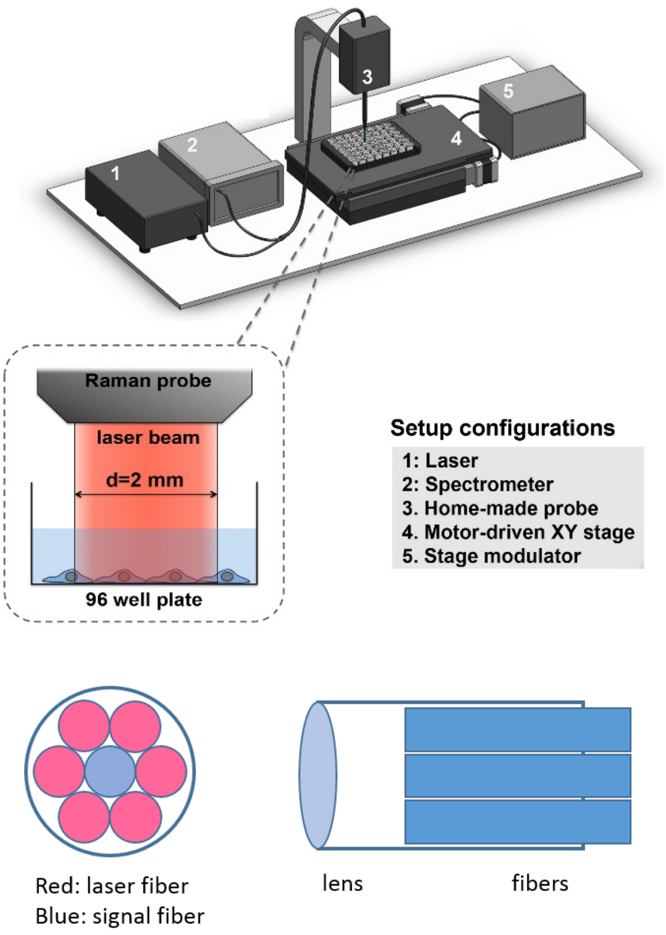

Figure S3. The setup configurations of the Raman plate reader used in current work. A Y-shape multimode fiber bundle containing 7 of 200 μm fibers were used to deliver the excitation laser to sample and collect the back scattering Raman signals into a spectrometer. In detail, 6 of the 7 fibers surrounding were used to deliver the laser beam to the sample through a collimation lens, and the rest one fiber at the center collected the Raman signals and send them to a fiber spectrometer. A filter was placed before the pinhole of spectrometer to block the reflected laser beam, which allows only the Raman signals (and probably some fluorescence signals) to pass through and be detected by the spectrometer. The output power of the excitation light was 500 mW, the power density at sample surface was roughly ~10^2 W/cm^2.

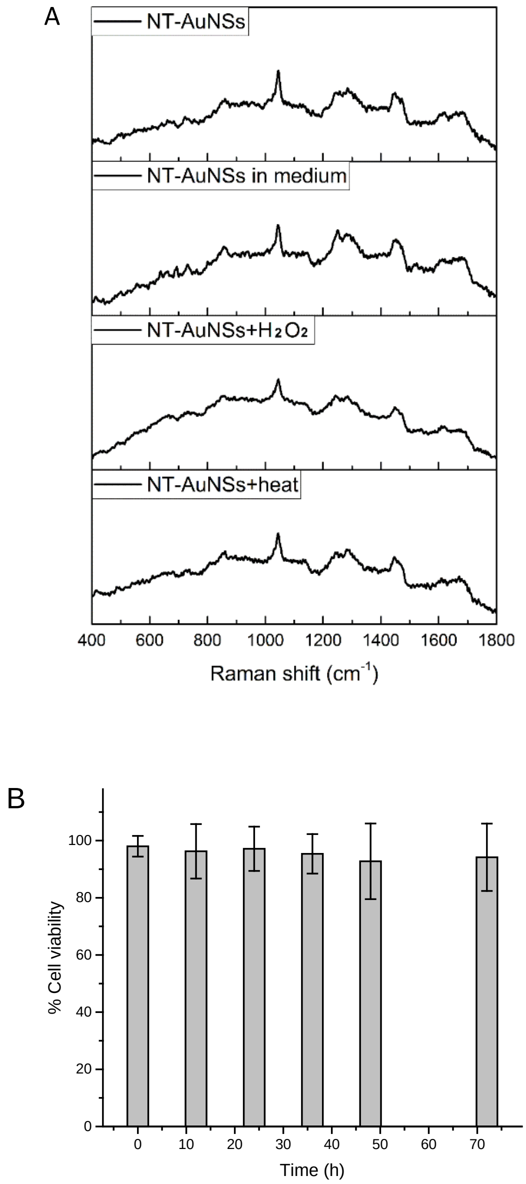

Figure S4. (A)Raman spectra of NT-AuNSs, NT-AuNSs incubated in cell culture medium for 24 h, NT-AuNSs treated by 1 mM H_2O_2 for 24 h and NT-AuNSs heated at 100 °C for 15 min. (B) XTT results of cell viability in the presence of 0.05 nM NT-AuNSs at different periods of incubation.

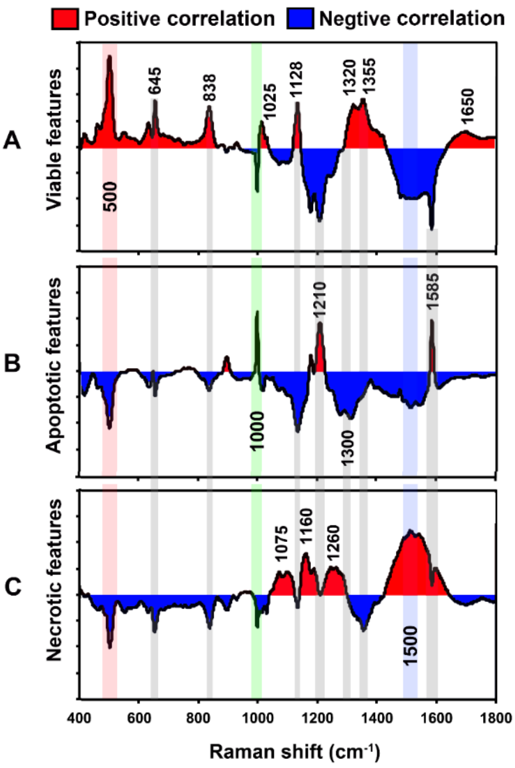

Figure S5. Representative PERS features of the viable (A), apoptotic (B) and necrotic cells (C).

Table S1. Analysis on the band intensity and representativity of PERS features originating from viable, apoptotic and necrotic cells.

PERS features	Feature intensity	Positive (+) or Negative (-)		
		Viable	Apoptotic	Necrotic
500	H	+	-	-
645	M	+	-	-
838	M	+	-	-
1000	H	-	+	-
1025	L	+	-	-
1075	L	-	-	+
1128	M	+	-	NA
1160	M	-	-	+
1210	H	-	+	NA
1260	L	-	-	+
1300	M	NA	-	NA
1320	M	+	-	-
1355	M	+	-	-
1500	H	-	-	+
1585	H	-	+	+
1650	L	+	-	-

Abbreviation: H=high; M=middle; L=low;
NA=not available

The band intensity H, M and L were defined according to the normalized intensity:

H: 100%-70%

M: 70%-30%

L: 30%-0

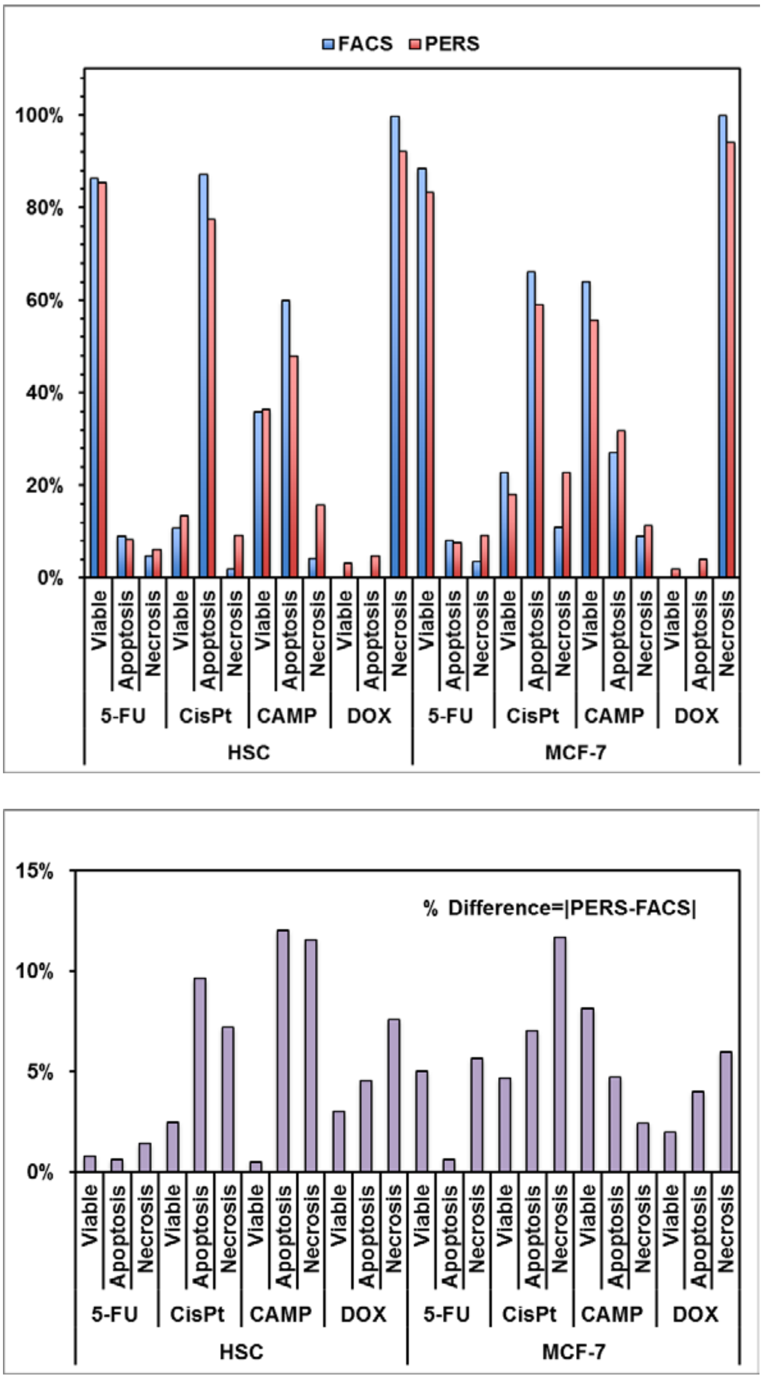

Figure S6. Comparison between results of FACS and PERS calculated according to the intensity of bands at 500, 1000 and 1585 cm^{-1}.

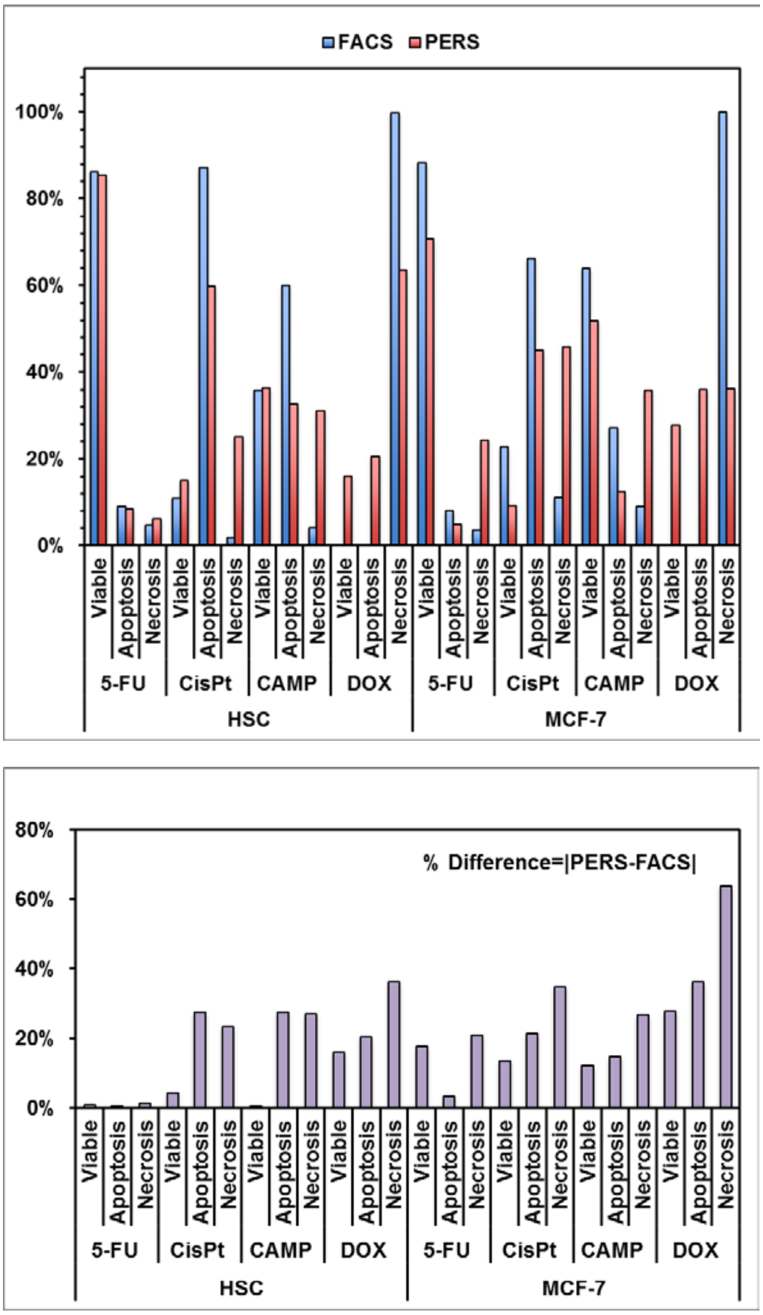

Figure S7. Comparison between results of FACS and PERS calculated according to the intensity of bands at 1180, 1210 and 1585 cm^{-1}.

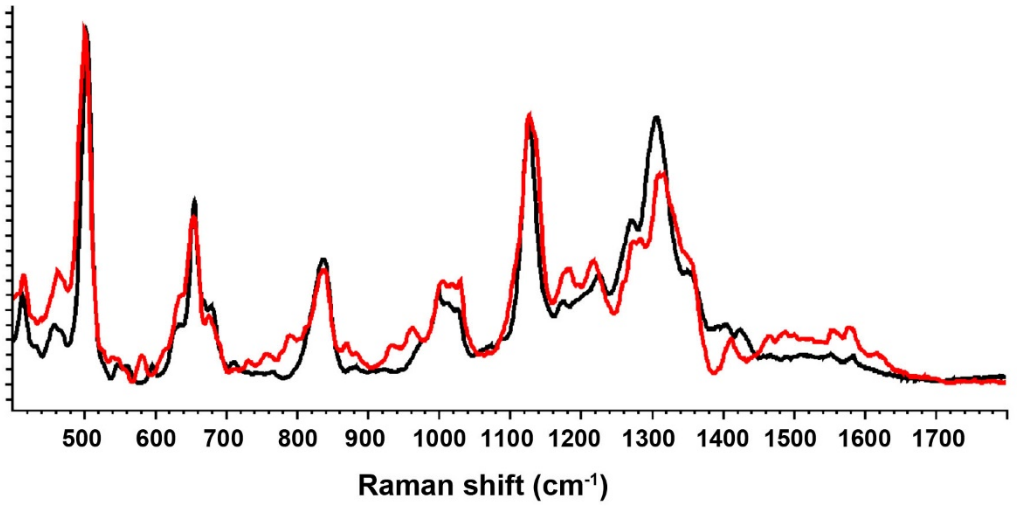

Figure S8. Plasmonic-enhanced Raman spectra of viable HSC-3 cells (black) and MCF-7 cells (red).

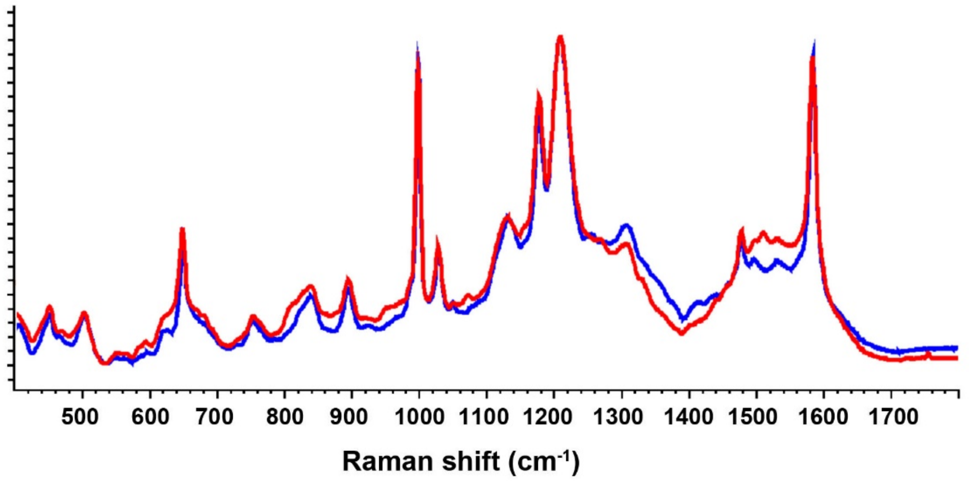

Figure S9. Plasmonic-enhanced Raman spectra of apoptotic HSC-3 cells (blue) and MCF-7 cells (red).

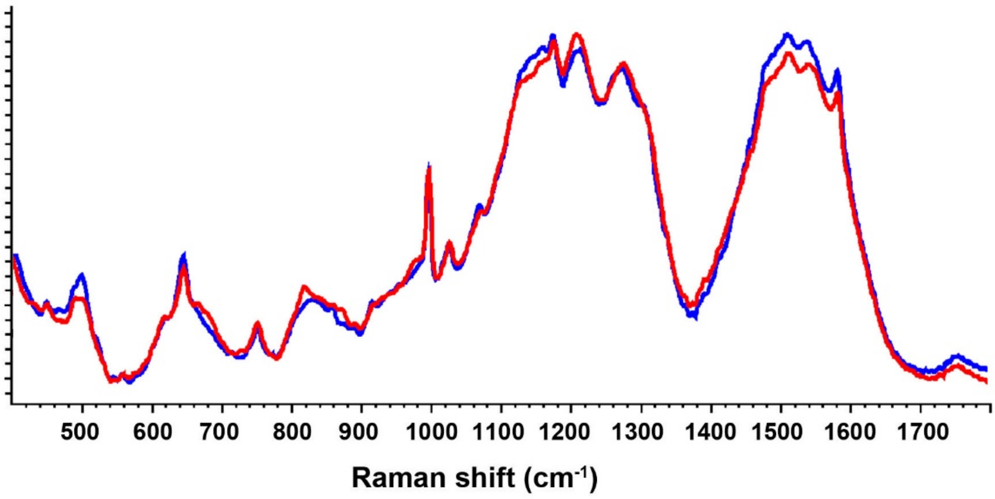

Figure S10. Plasmonic-enhanced Raman spectra of necrotic HSC-3 cells (blue) and MCF-7 cells (red).
